# Supplementary figures and images for: Endocytic myosin-1 is a force-insensitive, power-generating motor
Source: J Cell Biol. 2023 Aug 7;222(10):e202303095. doi: 10.1083/jcb.202303095 (PMC10406613; doi:10.1083/jcb.202303095)

Source Data: Figure 2

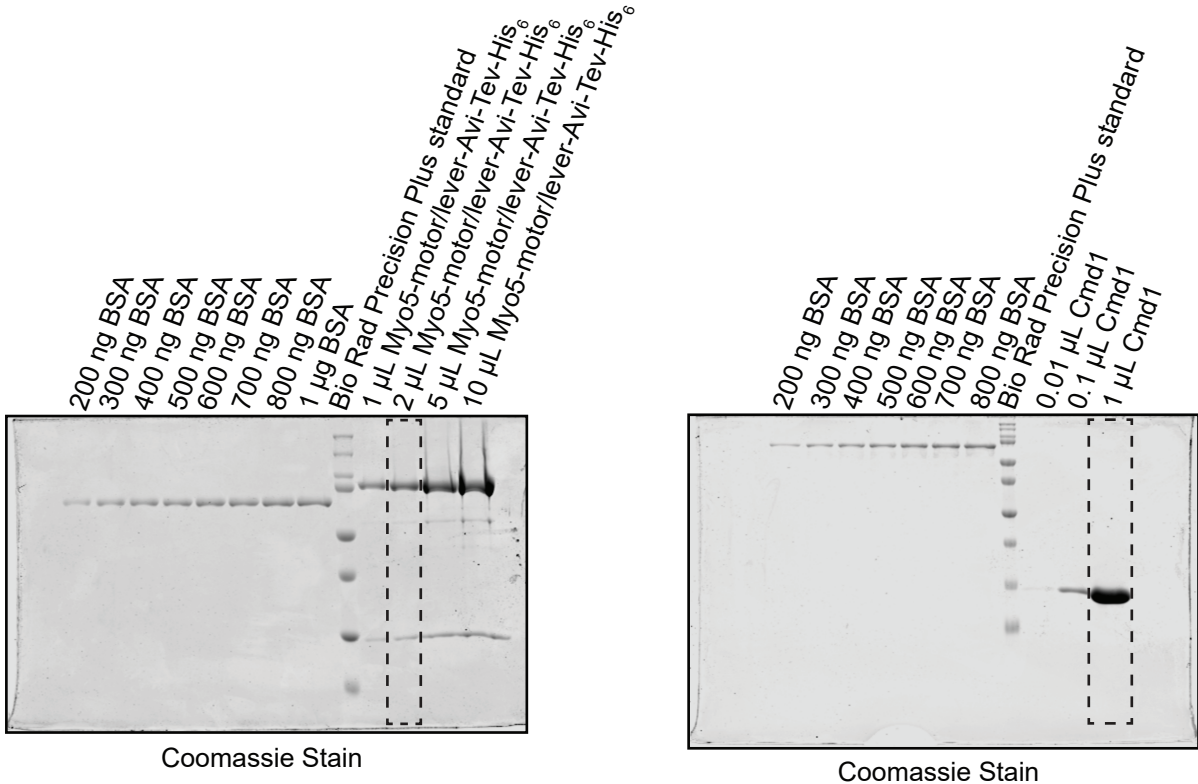

Supplement: SourceData F2 — is the source file for Fig. 2. [file JCB_202303095_SourceDataF2.pdf]
